# Natural products for the treatment of allergic rhinitis: focus on cellular signaling pathways and pharmacological targets

**DOI:** 10.3389/fphar.2024.1447097

**Published:** 2024-09-30

**Authors:** Shabnam Moradi, Hosna Khazaei, Mitra Tarlan, Seyed Vahid Jasemi, Tanuj Joshi, Ina Yosifova Aneva, Mohammad Hosein Farzaei, Javier Echeverría

**Affiliations:** ^1^ Pharmaceutical Sciences Research Center, Health Institute, Kermanshah University of Medical Sciences, Kermanshah, Iran; ^2^ Department of Internal Medicine, Kermanshah University of Medical Sciences, Kermanshah, Iran; ^3^ Department of Pharmaceutical Sciences, Bhimtal, Kumaun University (Nainital), Bhimtal, Uttarakhand, India; ^4^ Institute of Biodiversity and Ecosystem Research, Bulgarian Academy of Sciences, Sofia, Bulgaria; ^5^ Departamento de Ciencias del Ambiente, Facultad de Química y Biología, Universidad de Santiago de Chile, Santiago, Chile

**Keywords:** allergic rhinitis, phytochemicals, antioxidants, anti-inflammatory, signaling pathways, pharmacological targets

## Abstract

**Background:**

Allergic rhinitis is an inflammatory disease dependent on immunoglobulin E and causes inflammation of the nasal mucosa, leading to decreased quality of life for affected patients. Since common treatments, including corticosteroids and antihistamines, have temporary therapeutic effects and numerous side effects, investigating natural compounds effective in improving allergic rhinitis with low complications and high efficacy can be significant and necessary.

**Purpose:**

This study aims to present a comprehensive and critical evaluation of the effect of natural compounds in improving allergic rhinitis.

**Methods:**

Studies were identified through systematic searches of ScienceDirect, PubMed, Scopus, and Web of Sciences databases. Eligibility checks were conducted based on predefined selection criteria. Forty-six articles were included in this study.

**Results and discussion:**

Phytochemicals, including flavonoids, alkaloids, terpenoids, and other compounds showed significant anti-inflammatory and antihistaminic effects. These compounds alleviate allergic rhinitis symptoms by inhibiting inflammatory mediators, oxidative stress, apoptosis, and key signaling pathways such as MAPK/NFκB and TLR4/MyD88/NF-κB.

**Conclusion:**

Phytochemicals exhibit anti-inflammatory and antioxidant properties, making them.

## 1 Introduction

### 1.1 Definition, general introduction, and epidemiology of allergic rhinitis

Allergic rhinitis (AR) is characterized by inflammation of the nasal mucosa caused by exposure to an allergen (Varshney and Varshney, 2015). Exposure to allergens results in inflammation via the action of immunoglobulin E (IgE) antibodies. Some agents that can induce AR include animal dander, pollen, molds, and dust mites (Skoner, 2001). Asthma is worsened by AR and the majority of individuals with asthma experience AR (Liva et al., 2021). AR leads to a decrease in work and quality of life and imposes a monetary burden on various countries around the world (Nur Husna et al., 2022). There are two types of AR: seasonal and perennial. Seasonal rhinitis typically occurs during a specific season when the concentration of allergens in the air is high. Symptoms of seasonal rhinitis come and go quickly and can be easily detected based on exposure to allergens (Skoner, 2001). However, identifying perennial rhinitis can be challenging, as it often overlaps with other conditions such as vasomotor rhinitis, respiratory infections, and sinusitis. Perennial rhinitis is a type of AR that persists throughout most of the year (Bradding et al., 1993; Skoner, 2001). The allergic response associated with AR has a strong genetic link that is mediated by the activation of eosinophils, plasma cells, mast cells, and mucosal infiltration (Skoner, 2001). AR is first diagnosed based on specific clinical symptoms, as previously discussed. Following diagnosis, laboratory tests were conducted to identify specific allergens that trigger the production of IgE antibodies (Nur Husna et al., 2022). Laboratory tests, such as the skin prick test (SPT; percutaneous), *in vitro* serum allergen-specific IgE (ssIgE) immunoassay, and intradermal (intracutaneous) skin tests (IDST), are used to detect allergens in AR (Nam and Lee, 2017; Wongpiyabovorn et al., 2018).

There has been a significant rise in the number of cases of AR since 1990 (Wheatley and Togias, 2015). Approximately 40% of the adult population and 25% of children worldwide are affected by AR worldwide. The majority of the symptoms of AR develop before a person turns 20 years old (Skoner, 2001). The peak of these symptoms is seen between the ages of 20 and 40 years, and they eventually start declining (Wheatley and Togias, 2015). Studies have shown a high incidence of males suffering from AR in children, while females have a higher chance of experiencing AR in adolescence (Fröhlich et al., 2017; Pinart et al., 2017). The prevalence of AR has rapidly increased over the years due to increased industrialization and pollution (Nur Husna et al., 2022). Seasonal AR is more prevalent in children than in adults, whereas perennial rhinitis is more common in adults. Approximately one-fifth of people with rhinitis later develop asthma. There is significant geographical variation in the prevalence of AR, asthma, and other similar diseases (Varshney and Varshney, 2015). The prevalence of AR is higher among individuals with higher socioeconomic status, those living in polluted areas, non-white people, individuals with a family history of allergies, and those born during the pollen season. First-born children are at a higher risk of developing AR. Studies in children during the first few years of life have revealed that several factors can increase the risk of developing AR. These factors include heavy smoking by their mothers in the first year of their lives, early exposure to food or formula, exposure to allergens found inside the house such as dust mites and animal dander, allergic complications in parents, and high serum levels of IgE (Bousquet et al., 2020).

### 1.2 Allergic rhinitis complications

AR causes inflammation of the nasal membrane. Symptoms of rhinitis can often be distressing. It can lead to a depressed mood, fatigue, sleep disturbances, and cognitive function problems. All these symptoms have a devastating effect on both quality of life and work (Meltzer, 2001). In addition, the aforementioned complications, otitis media, conjunctivitis, postnasal drip, dysfunction of the eustachian tube, and sinusitis are additional complications. Facial deformities and dental malocclusions can occur in children (Bousquet et al., 2006; Varshney and Varshney, 2015). Nasal blockage, sneezing, nasal itching, tearing, redness, and eye itching are the symptoms experienced by patients with AR. Coughing and itching of the palate also occur (Bousquet et al., 2008; Brożek et al., 2017; Nur Husna et al., 2022). Allergic complications can weaken the body in certain patients and may lead to severe reactions, such as anaphylaxis (Valenta et al., 2018).

### 1.3 Cellular and molecular mechanisms involved in allergic rhinitis

Various cellular and molecular mechanisms, including multiple pathways, are involved in the induction and progression of AR. Type 1 helper (Th1) and type 2 helper (Th2) cells play significant roles in AR. Studies have shown that recruitment and activation of IgE antibody-producing B cells, mast cells, and eosinophils are mediated by Th2 cells. Delayed-type hypersensitivity and interferon-γ (IFNγ) production play a major role in Th1 cells. IFNγ plays an important role in destroying phagocytosed microbes at the intracellular level (Bellanti, 1998; Deo et al., 2010). Regulation of eosinophil and basophil-mediated responses and secretion of interleukin (IL)-4, IL-5, IL-6, and IL-13 are important functions of Th2 cells (Malmhäll et al., 2007). Many studies have suggested that in AR, there is a relative deficiency of Th1 cells on one hand and an upregulation of Th2 cells. This theory explains many aspects of AR, but not all. Many other mechanisms are also involved in the development of AR (Reisinger et al., 2005; Malmhäll et al., 2007). With the advancement of science and the discovery of regulatory T (Treg) and Th17 cells, new dimensions have been added to the original Th1/Th2 balance theory (Wang et al., 2014). Currently, the Th9 cell subset plays a significant role in allergic responses. This subset is important because it shows a high expression of IL-9 (Cortelazzi et al., 2013; Esmaeili and Alilou, 2014; Schlapbach et al., 2014). Normal tissues and inflammatory cells are affected by IL-9. IL-9 plays a crucial role in various processes, such as increasing the number of mast cells, eosinophils, and lymphocytes. It also stimulates the secretion of IgE, enhances mast cell responses to allergens, promotes mucin expression, and stimulates cytokine secretion by inflammatory cells (Dugas et al., 1993; McLane et al., 1998; Louahed et al., 2000; Soussi-Gounni et al., 2001). Although Th9 cells are involved in allergic asthma, their exact role in AR remains unclear. Inflammatory responses are regulated by various subsets of T cells via secretion of cytokines such as IL-4, IL-5, IL-9, IL-13, IL-17, and IFN-γ (Kaplan, 2013). Several studies have shown that the presence of cytokines such as IL-1, IL-2, IL-4, IL-6; tumor necrosis factor α (TNFα); granulocyte-macrophage colony-stimulating factor (GM-CSF), and sargramostim in nasal secretions is involved in the development and progression of AR (Fireman, 1996; Verschoor and von Gunten, 2019). Various pathways are involved in the development of AR. Nuclear factor-kappa B (NF-κB) plays a crucial role in numerous inflammatory and immune processes. It does so by regulating the cytokines involved in the immune and inflammatory responses. In addition, it regulates the expression of inflammatory mediators (Ghosh et al., 1998). Although the role of NF-κB in asthma has been established, clear information regarding its role in AR is not yet available. It has been demonstrated in both animal and human studies that NF-κB is upregulated in the nasal mucosa of patients with AR (Kim et al., 2000; Wang and Zheng, 2011; Wang et al., 2013; Wee et al., 2017). There is also a link between Toll-like receptors (TLRs) and the NF-κB pathway in AR. TLRs play a crucial role in allergic transduction pathways. TLRs are important components of the immune system (He and Wang, 2021). Enhanced protein and mRNA expression of TLR-2 and TLR-4 have been demonstrated in patients with persistent AR (Cui et al., 2015). In addition, high expression of TLR3, TLR7, and TLR9 has been detected in the nasal epithelial cells of patients with AR. Initially, when TLRs were activated, they exhibited a protective response. However, constant activation of TLRs leads to an inflammatory response owing to the continuous release of proinflammatory chemokines and cytokines (Matsushima et al., 2004). TLRs induce the activation of NF-κB, which in turn produces important pro-inflammatory effects (Zhang et al., 2014). Mitogen-activated protein kinases (MAPKs) also play an important role in AR. In an experimental study, it was found that p38 MAPK plays a role in AR, specifically in the release of Th2 cytokines (Liu et al., 2010). It has been observed that MAPKs are involved in the expression of pro-inflammatory genes (Lasa et al., 2001). Inhibition of the MAPK pathways results in increased susceptibility of proinflammatory gene mRNA to rapid breakdown. This leads to a decrease in the expression of these genes (Barnes, 2006). An experimental study has shed light on the association between the extracellular signal-regulated protein kinase (ERK) signaling pathway in CD4+T cells and the development of AR (Ai et al., 2021). Some researchers have explored the role of oxidative stress in the development of AR. An imbalance between oxidative species and the body’s antioxidant defense system often leads to various physiological and pathological conditions. This imbalance often leads to conditions such as chronic inflammation (Pawankar et al., 2011; Lim J.-O. et al., 2021). According to the literature, AR is characterized by an excessive formation of reactive oxygen species (ROS), which inhibits the body’s antioxidant system and triggers inflammatory responses (Sugiura and Ichinose, 2008; Pawankar et al., 2011; Lim J.-O. et al., 2021; Lim et al., 2021 S.).

### 1.4 Restriction of current treatments and the role of natural products in allergic rhinitis

Currently, the therapeutic approaches to treating AR include avoidance (i.e., avoiding allergens or allergic conditions to which a patient is susceptible), immunotherapy, and pharmacotherapy. Patients should be kept in an environment and social conditions that positively maintain their quality of life and work efficiency (Varshney and Varshney, 2015). The fundamental concept behind allergen immunotherapy is that relevant allergens are administered to the patient through subcutaneous injection. The dose of the allergen was gradually increased until an effective dose was reached to induce immunological tolerance to the allergen. Allergen immunotherapy is an effective treatment for AR, especially in patients with seasonal AR caused by pollen (Calderon et al., 2007; Frew, 2010; Walker et al., 2011; Small et al., 2018). This therapy is also effective against AR caused by dog and cat dander, dust mites, and cockroaches. Allergen immunotherapy should only be considered if a patient is unresponsive to conventional treatments, as this therapy carries the risk of anaphylaxis, a potentially life-threatening reaction. In addition, therapy should only be carried out under the supervision of a physician who has experience in treating allergies and possesses adequate equipment to manage anaphylaxis. Anaphylaxis is a severe adverse effect associated with allergen immunotherapy (Small et al., 2007). The therapy shows beneficial effects only when administered for a minimum of 3 years, and these effects can last for several years (Durham et al., 1999; Eng et al., 2006). Allergen immunotherapy also offers protection to children with AR against the future development of asthma in the future (Frew, 2010). Sublingual immunotherapy, which involves placing a tablet containing an extract of an allergen under the tongue until it dissolves, is also used to treat AR. The local side effects associated with this route include ear pruritus, throat irritation, and oral pruritus (Small et al., 2018). In pharmacotherapy for AR, the treatment is administered using a variety of drugs available on the market. Nasal saline irrigation solutions, intranasal corticosteroids, oral antihistamines, a combination of corticosteroid/antihistamine sprays, and leukotriene antagonists have been used to treat AR (Small et al., 2018). Histamine blockers, such as cetirizine, loratadine, desloratadine, and fexofenadine, are used for the treatment of AR. Recently, rupatadine and bilastine were introduced for the treatment of AR (Durham et al., 1999). Zafirlukast and montelukast are leukotriene antagonists that are effective in the treatment of AR. However, they are not as effective as intranasal corticosteroids (Pullerits et al., 1999). They are primarily used when antihistamines or corticosteroids are not well tolerated or are ineffective in managing AR (Small et al., 2007; Kim and Kaplan, 2008). Intranasal corticosteroids are crucial primary medications for treating AR. Studies have shown that intranasal corticosteroids are more effective than antihistamines and leukotriene antagonists in treating the symptoms of AR (Weiner et al., 1998; Pullerits et al., 2002; Yáñez and Rodrigo, 2002; Wilson et al., 2004). Various corticosteroids such as beclomethasone, fluticasone propionate, triamcinolone acetonide, fluticasone furoate, ciclesonide, mometasone furoate, and budesonide have been used to treat AR. The proper use of intranasal sprays is crucial for the effective delivery of corticosteroids. Therefore, it is essential to provide patients with proper advice on how to administer intranasal corticosteroid sprays. It is ideal to start using intranasal corticosteroid sprays just before exposure to allergens because it takes several days for their peak effect to develop. They are used daily by patients with AR (Lee and Mace, 2009). If intranasal corticosteroids are ineffective on their own, they can be used in combination with antihistamines. This combination is more effective than the individual drugs. Combinations such as fluticasone propionate–azelastine (which acts within minutes) and mometasone–olopatadine (which acts in approximately 1 h) are available for use (Hampel et al., 2010; Bousquet et al., 2018; Patel et al., 2019; Segall et al., 2019). Corticosteroids have various adverse effects such as stinging, nasal irritation, and epistaxis. These adverse effects can be prevented by using a spray containing corticosteroids slightly away from the nasal septum (Small et al., 2007; Wu E. L. et al., 2019). Intranasal corticosteroids, such as triamcinolone and beclomethasone, may cause growth retardation in children compared with placebo. Other corticosteroids, besides those mentioned above, do not cause growth retardation (Agertoft and Pedersen, 2000; Schenkel et al., 2000; Skoner, 2001; Allen et al., 2002). Based on the aforementioned observations, it can be said that the current therapy for AR focuses on treating the symptoms of the condition. Medications must be taken regularly; however, there is no complete relief from the condition. However, we can also explore the natural benefits of medicinal plants and natural products for treating AR. Some medicinal plants have traditionally been used to treat AR; however, further scientific research is needed to establish their effects. Many natural products have anti-inflammatory effects and regulate various mediators of AR. They also act via their antioxidant activity and control the activity of the NF-κB pathway, which is stimulated by ROS. IL-4, IL-5, IL-13, TNF-α, IFN-γ, cyclooxygenase 2 (COX-2), and phospho-ERK1/2 (p-ERK1/2) are the main mediators associated with AR and can be influenced by natural products (Lim S. et al., 2021). Medicinal plants and natural products have been demonstrated to be effective in reducing inflammation, sneezing, and other symptoms associated with AR (Lim S. et al., 2021; Rahim et al., 2021). Clinical trials have demonstrated the effectiveness and safety of natural products for the treatment of AR. Natural products have the advantage that, when used with proper information and in suitable doses, they can prove to be better tolerated and safer than current allopathic treatments. In the future, newer bioactive and other natural products may be discovered that can offer long-term relief or even a complete cure for AR (Lim S. et al., 2021; Rahim et al., 2021).

## 2 Methods

### 2.1 Search strategy

**FIGURE 1:**
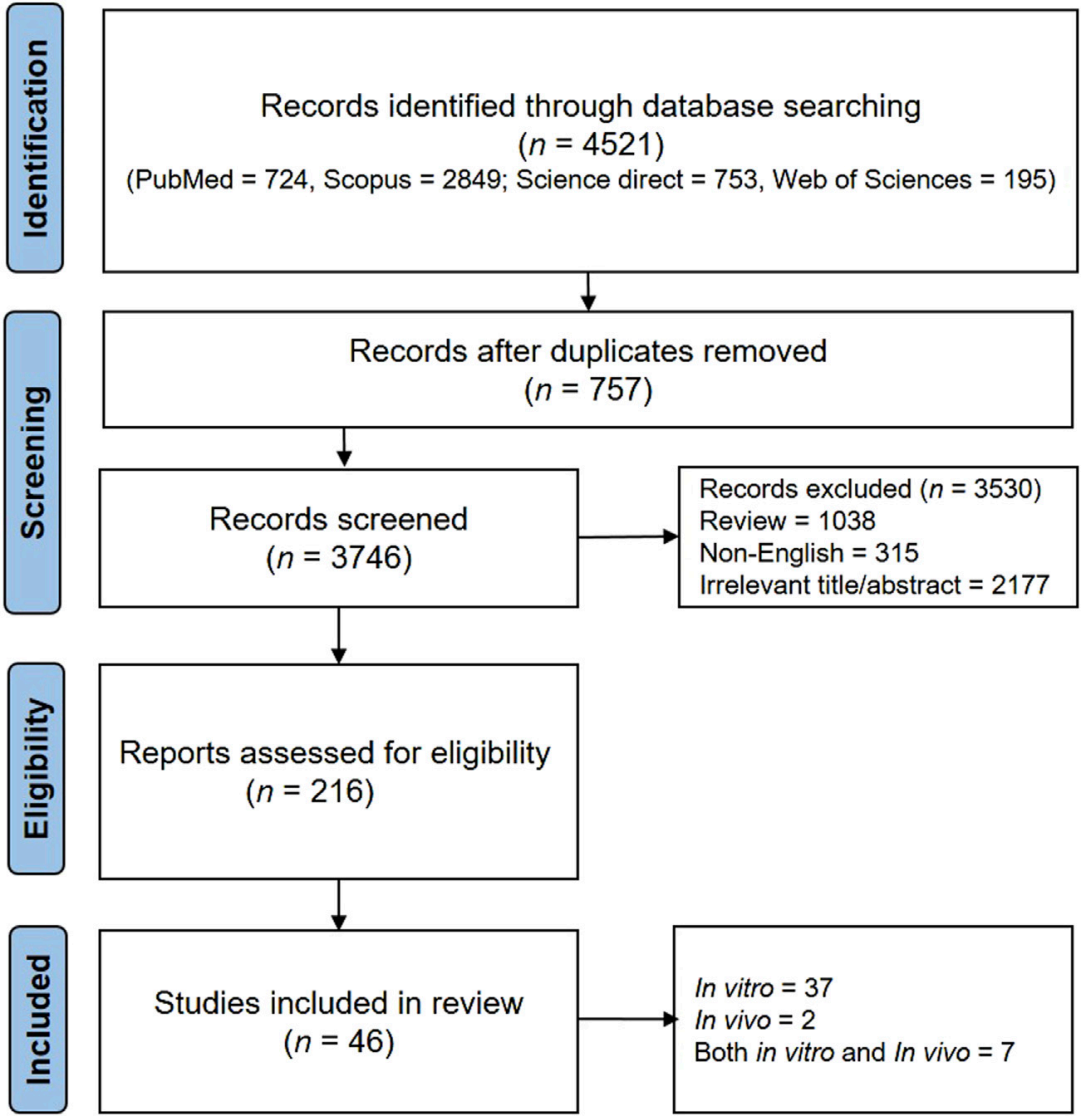
The PRISMA flowchart on the literature search procedure and selection of related studies.

The present systematic review adhered to the guidelines outlined in the Preferred Reporting Items for Systematic Reviews and Meta-Analysis (PRISMA) (Moher, 2009). Multiple electronic databases (Science Direct, PubMed, Web of Sciences, and Scopus) were used. The search was conducted using the keywords “allergic rhinitis” in the title/abstract and “plant” OR “herb” OR “phytochemical” OR “polyphenol” OR “phenolic” OR “flavonoid” OR alkaloid” in the full text. Additional information regarding the selection of articles can be found in the PRISMA diagram in Figure 1.

### 2.2 Inclusion criteria

Experimental studies (*in vivo* and *in vitro*) in the English language that assessed the effects of natural products on AR were included.

### 2.3 Exclusion criteria

We implemented the following criteria for exclusion: 1) conference abstracts, books, book chapters, and unpublished findings; 2) papers not written in English; 3) reviews, systematic reviews, meta-analyses, and letters; and 4) primary research papers that did not utilize tumor cell lines or animal models.

### 2.4 Data extraction

The final eligible studies were reviewed for phytochemical name, study design, model, intervention, and outcome. The separation information of the articles is visible in the PRISMA diagram (Figure 1).

## 3 Results

In this study, 4,521 results were obtained, of which 757 studies were excluded due to duplication. After that, 315 studies were excluded because they were not in English, 1,038 were excluded because they were reviews, and 2,177 were excluded because the title and abstract were irrelevant. Finally, 46 articles were examined entirely and included in the present study. In the following, we have reviewed the valuable information from these articles.

### 3.1 Alkaloids and nitrogen-containing compounds

#### 3.1.1 Berberine

Berberine (5,6-Dihydro-9,10-dimethoxybenzo[g]-1,3-benzodioxolo[5,6-a]quinolizinium) a quaternary ammonium salt from the protoberberine group of benzylisoquinoline alkaloids is usually found in *Berberis* species (Neag et al., 2018). Berberine can decrease blood IgE levels, tissue eosinophil counts, GATA-3 mRNA levels, T-bet mRNA levels, and allergic inflammation in a mouse model of AR. Furthermore, berberine increased the proportion of CD4^+^ CD25^+^ Foxp3+ T cells, indicating its potential role in the control of Treg cells (Kim et al., 2015).

#### 3.1.2 Dictamenine

Dictamenine is a furoquinoline alkaloid found in the medicinal plant *D. dasycarpus* Turcz. [Rutaceae]. Dictamenin has shown its ability to inhibit the molecular signaling pathway mediated by LYN kinase during the activation of mast cells, which plays an important role in the early stages of the pathogenesis of AR. Dictamanin has also been shown to reduce nasal rubbing and sneezing in a murine model of ovalbumin (OVA)-induced AR (Liu R et al., 2023). Furthermore, an analysis of the results of 19 randomized controlled trials has shown that Chinese herbal medicines, including *D.dasycarpus*, are more effective in treating AR in children than in the control groups (Zheng et al., 2018). However, it must be mentioned that Cortex dictamni, which includes *Dictamnus dasycarpus*, is associated with potential hepatotoxicity (Fan et al., 2018).

#### 3.1.3 Ellipticine

Ellipticine (5,11-dimethyl-6h-pyrido(4,3-b)carbazole) is a pyridindole alkaloid discovered in the leaves of some *Ochrosia* species (Miller and McCarthy, 2012). Several studies have demonstrated its potential as an antitumor agent, showing favorable characteristics with limited side effects and no hematotoxicity (Shvydenko et al., 2022). Ellipticine was investigated for its potential protective properties in murine models of OVA-induced AR. The findings show that ellipticine can reduce allergic inflammatory responses and symptoms associated with AR through the dual inhibition of COX-2 and NF-κB (Wang et al., 2021).

#### 3.1.4 Higenamine

Higenamine ((*S*)-Norcoclaurine) is a benzyltetrahydroisoquinoline alkaloid that has various effects such as relaxing blood vessels and trachea and having antioxidant, anti-apoptotic, anti-inflammatory, and immune system-modulating properties. A study investigated the effect of higenamine and its underlying mechanism on AR. According to the study, higenamine may reduce AR by inhibiting EGFR/JAK2/c-JUN signaling and activating AKT1. This study also showed that higenamine affects histamine one-induced changes in serine/threonine kinase 1 (AKT1), EGFR, c-Jun, iNOS, and JAK2 (Wei et al., 2021).

#### 3.1.5 *N,N*-dicoumaroylspermidine

*N,N*-dicoumaroylspermidine is a phenolic amine derived from *Lithospermum erythrorhizon* Siebold & Zucc. [Boraginaceae] that exhibit significant antiallergic properties. In a study using an OVA-induced AR mouse model, *N,N*-dicoumarolespermidine reduced serum OVA-specific IgE production and the abundance of inflammatory cells in nasal lavage fluid. These findings suggest that *N,N*-dicoumarolespermidine is a promising therapeutic agent for AR (Le et al., 2022).

#### 3.1.6 Piperine

Piperine (1-peperoyl piperidine) is a simple and pungent piperidine-type alkaloid found in the seeds of black pepper (*Piper nigrum* L. [Piperaceae]) that shows many pharmacological activities, including anti-oxidant, anti-inflammatory, immune modulator, anti-cancer, and anti-asthmatic activities (Chopra et al., 2016). Aswar et al. demonstrated that the treatment of AR mice with piperine reduced spleen weight and reduced NO, histamine, IL-1β, IgE, and IL-6 levels in the serum (Aswar U. et al., 2015). Therefore, this phytochemical, which reduces oxidative stress and suppresses the production of inflammatory cytokines, modulates AR activity.

#### 3.1.7 Sinomenine

Sinomenine is an isoquinoline-type alkaloid derived from *Sinomenium acutum* (Thunb.) Rehder and E.H.Wilson [Menispermaceae]. The anti-inflammatory and immunomodulatory activities of this herb have been previously confirmed (Lodge et al., 1974; Yamasaki, 1976). Chen et al. demonstrated that AR mice treated with sinomenine showed a reduction of rubbing and sneezing also reduction in the eosinophils levels in the nasal mucosa, a reduction in the IgE, IL-4, and IFN-ϒ levels in the serum, and an increase in the TGF-β expression in the serum and nasal mucosa (Chen et al., 2017). Therefore, sinomenine improves AR by modulating Th2 cytokine and eosinophil infiltration.

#### 3.1.8 Warifteine

Warifteine, an bisbenzylisoquinoline-type alkaloid discovered in *Cissampelos sympodialis* Eichler [Menispermaceae], has shown the ability to modulate the allergic profile in a model of chronic AR. In particular, it has been shown to reduce immediate allergic reactions and thermal hyperalgesic responses in sensitized animals. In a specific study, inhaled warifteine at a concentration of 2 mg/mL was used to treat OVA-sensitized BALB/c mice with chronic AR. The results showed a decrease in the frequency of sneezing and nose rubbing as well as a decrease in total IgE and OVA-specific IgE levels in the bloodstream. These findings suggest that warifteine may have a beneficial effect on AR by reducing allergic responses (Vieira et al., 2018).

### 3.2 Flavonoids

#### 3.2.1 Apigenin

Apigenin (4′,5,7-Trihydroxyflavone) is a bioactive plant flavone with anti-allergic properties. Apigenin reduces AR inflammation by inhibiting the activation of the TLR4/MyD88/NF-κB signaling pathway. Apigenin also reduced allergic reactions to OVA-induced AR in laboratory mice by modulating Th1/Th2 responses. In addition, apigenin modulates the Th1/Th2 balance by suppressing Th2 responses (IgE, histamine, ILs, GATA3, Signal transducer and activator of transcription 6 (STAT6), Suppressor of cytokine signaling-1 (SOCS1), and NF-κB) (Chen et al., 2020).

#### 3.2.2 Baicalin

Baicalin is the main glycosyloxyflavone of *Scutellaria baicalensis* Georgi [Lamiaceae]. Its pharmacological activities have been confirmed including anti-oxidant, anti-inflammatory, anti allergic (Zhou et al., 2016). Chen et al. showed that treatment of AR rats with baicalin reduced IgE, histamine, IL-4, IL-13, IL-6, and TNF-α levels in serum and also reduced nasal sneezes in behavioral tests (Chen et al., 2019).

#### 3.2.3 Cirsilineol

Cirsilineol (4′,5-Dihydroxy-3′,6,7-trimethoxyflavone) is a bioactive flavone with anti-inflammatory and anti-allergic properties that can protect against OVA-induced AR, according to a study in mice. This study showed that administration of cirsilineol effectively reduced sneezing and nasal rubbing in OVA-stimulated mice and reduced the levels of IgE, prostaglandin D2 (PGD2), and leukotriene C4 (LTC4) in animals with AR. These findings suggested that cirsilineol could potentially serve as a therapeutic option for AR (Li et al., 2021).

#### 3.2.4 Diosmetin

Diosmetin (5,7,3′-trihydroxy-4′-methoxyflavone), a natural *O*-methylated flavone found in tangerines, exhibits significant anti-inflammatory and antioxidant properties (Lee et al., 2020). Hu and Peng showed that diosemetin effectively reduced OVA-induced nasal inflammation in mouse models of AR by regulating the sirtuin 1 (SIRT1)/NF-κB signaling pathway (Hu and Peng, 2023).

#### 3.2.5 Hesperidin

Hesperidin (Hesperetin, 7-rutinoside) is a flavonoid glycoside often found in citrus fruits, including grapefruit, oranges, and lemons. Hesperidin have anti-inflammatory and antioxidant properties, they can be used to treat a variety of inflammatory conditions, including AR. Hesperidin was studied to determine how they affect mice with OVA1-induced AR. According to this study, hesperidin reduces inflammation and allergy symptoms in AR. Comparing the AR group with the other groups, a significant decrease in total antioxidant capacity (TAC) levels and an increase in IgE, IL-5, IL-13, and total oxidant status (TOS) levels were observed. There was a significant increase in these parameters for hesperidin group (Kilic et al., 2019).

#### 3.2.6 Kaempferol

Kaempferol (3,4′,5,7-tetrahydroxyflavone) is a naturally occurring flavonol found in fruits and vegetables that shows pharmacological activities such as anti-oxidant, anti-cancer, anti-inflammatory, and anti-asthma effects (Molitorisova et al., 2021; Sharma et al., 2021). Oh et al. demonstrated that *in vitro* treatment of Eosinophilic leukemia cell line-1 cells (EoL-1) with kaempferol reduced IL-8 levels and Caspase-1 activity. *In vivo*, treatment of AR mice with kaempferol reduced spleen weight, IgE, and histamine levels in serum, reduced IL-4, and increased IFN-ϒ levels in spleen tissue in nasal mucosa treated with kaempferol reduced IL-32, thymic stromal lymphopoietin (TLSP) expression, and MIP-2, ICAM-1 and Cox-2 levels (Oh et al., 2013). Therefore, kaempferol improves AR by regulating inflammatory markers.

#### 3.2.7 Luteolin

Luteolin (3′,4′,5,7-Tetrahydroxyflavone) is a flavone aglycone that is present in *Perilla frutescens* (L.) Britton [Lamiaceae], and exhibit a wide range of pharmacological activities, such as antioxidant, anti-inflammatory, anti-cancer, and immunomodulatory activities (Jeon et al., 2014). Liang et al. demonstrated that treatment of AR mice *in vivo* reduced IgE and IgG levels, increased IgG2α levels in the serum, increased IFN- and IL-17 cytokine levels, and reduced IL-4 and IL-10 levels in the spleen tissue. In an *in vitro* study on peripheral blood mononuclear cells from AR patients, treatment with luteolin blocks Th2 cytokine production via inhibition of STAT-6 and GATA-3 expression (Liang et al., 2020). Also, luteolin suppresses IL-4, a cytokine essential for AR development In mice with AR, luteolin effectively reduces mucus production, serum IgE levels, and allergic symptoms. In addition, luteolin promotes downregulation of the Th1/Th2 imbalance (Dong et al., 2021).

#### 3.2.8 Morin

Morin (3,5,7,2′,4′-pentahydroxyflavone), a pentahydroxyflavone is an important phytochemical in many plants belonging to the Moraceae family (e.g., *Morus alba* L. [Moraceae]). It has been found to have antioxidant, anti-inflammatory, anti-tumor, and neuroprotective properties that help in a variety of human diseases (Thakur et al., 2020). Liang et al. showed that morin administration in BALB/c mice has anti-allergic properties against OVA-induced AR in BALB/c one to two mice. Murine anti-allergic action is achieved by blocking the STAT6/SOCS1 and GATA3/T-bet one to two signaling pathways. Morin ameliorates OVA-induced AR by inhibiting the STAT6/SOCS1 and GATA3/T-bet signaling pathways in BALB/c mice (Liang et al., 2019).

#### 3.2.9 Myricetin

Myricetin (3,3′,4′,5,5′,7-Hexahydroxyflavone) is a common plant-derived flavonol with anti-inflammatory, anti-allergic, and antioxidant properties. According to a previous study, myricetin protects mice from AR caused by OVA by controlling the Th1/Th2 balance. In addition, myricetin significantly reduced nasal symptoms and histamine levels. However, there is no clear evidence for the effect of myricetin on AR in humans (Shi et al., 2023).

#### 3.2.10 Naringenin

Naringenin ((2*S*)-4′,5,7-Trihydroxyflavan-4-one), a flavanone found in citrus fruits, has been investigated for its potential protective properties against AR in rats. In a study by Sahin et al., rats with induced AR were orally treated with naringenin for 7 days. The group treated with naringenin showed significant clinical recovery, with lower sneezing and nasal itching scores than the AR group (Şahin et al., 2021). Additionally, the serum levels of total IgE, IL4, and IL5 were significantly lower in the naringenin group, indicating a decrease in allergic inflammation. Histopathological examination also revealed significant improvement in the nasal structures in the naringenin group.

#### 3.2.11 Quercetin

Quercetin (3,3′,4′,5,7-Pentahydroxyflavone) is the major flavonoid involved in vegetables and fruits and has many pharmacological activities, such as anti-inflammatory, anti-cancer, anti-oxidant, and immune modulators (Wang W. et al., 2016; Lucarini et al., 2021; Fakhri et al., 2022). Sagit et al. showed that the treatment of AR rats with quercetin inhibits inflammatory pathways through the inhibition of Cox-2 and vasoactive intestinal peptide (VIP) expression in the nasal tissue (Sagit et al., 2017). Cox-2 plays an important role in the inflammatory response, and VIP is a regulator of mucus secretion in the nasal mucosa (Shaida et al., 2001; Raap and Braunstahl, 2010).

#### 3.2.12 Skullcapflavone II

Skullcapflavone II (neobaicalein) is a flavone isolated from *S. baicalensis* Georgi [Lamiaceae] and shows pharmacological activities such as anti-inflammatory, immune modulator, anti-oxidant, anti-bacterial, anti-cancer, anti-allergy, and anti-asthma effects (Jung et al., 2012; Tsai et al., 2015). Bui et al. demonstrated that treatment of AR mice with skullcapflavone II reduced eosinophil levels, and PAS + cells and mast cells in the nasal tissue also reduced IgE and IgG1 levels in serum but had no significant effect on IgG2α levels, as well as reduced TNF-α, IL-4, IL-13, and GATA-3 levels and increased IL-12 levels in the nasal lavage fluid and lung tissue, but had no significant effect on IL-10 and IFN-levels. In addition, it reduced histamine and NF-κB and increased IκB levels in nasal lavage fluid and lung tissue (Bui et al., 2017). Therefore, this phytochemical modulates AR via inhibition of the NF-κB signaling pathway, histamine release, and Th2 cytokine release.

#### 3.2.13 Tangeretin

Tangeretin (4′,5,6,7,8-Pentamethoxyflavone) is a flavonoid found in the peels of tangerines and other citrus fruits. It has been found to have various medicinal properties, including anti-inflammatory effects. A mouse study revealed that tangeretin might enhance regulatory T cell responses by reducing Notch1/Jagged1 expression and increasing the growth of FOXP3/Treg cells, and thus could serve as a potential treatment for AR. Researchers investigated the effect of tangeretin on allergy symptom scores, OVA-specific IgE titers, histopathological features, and cytokine levels associated with T-helper cells (Th1, Th2, and Th17). The number of splenic CD4^+^ CD25^+^ FOXP3+ Treg cells and the transcription factor FOXP3 significantly increased when tangeretin was administered to AR mice or naïve CD4^+^ T cell development. This is followed by a concomitant decrease in Notch1/Jagged1 expression (Xu et al., 2019). In contrast, dexamethasone treatment of AR did not appear to be associated with Notch1/Jagged1 expression or Treg cell differentiation, which served as a positive control.

### 3.3 Phenolic compounds

#### 3.3.1 Gallic acid

Gallic acid, also known as 3,4,5-trihydroxybenzoic acid, is found in several herbs and fruits. The pharmacological activities of this phenolic acid including anti-oxidant, anti-inflammatory, anti-diabetes, and anti-cancer activities, have been confirmed (Kim et al., 2006). Fan et al. demonstrated that treatment of AR mice with gallic acid reduced the levels of eosinophils, neutrophils, lymphocytes, and macrophages in the nasal lavage fluid and reduced the IgE, IgG1, and IgG2α levels in the serum. Gallic acid treatment reduced IL-4, IL-5, IL-13, IL-17, and Related orphan nuclear receptor ϒt (RORϒt), and increased IFN-ϒ and IL-12 levels in the nasal lavage fluid. A reduction in rubbing and sneezing in mice confirmed these biochemical results (Fan Y. et al., 2019). This study confirmed that gallic acid improved AR by modulating the Th1/Th2 response.

#### 3.3.2 Proanthocyanidins polyphenols

Proanthocyanidin polyphenols (type A) derived from *Cinnamomum verum* J.Presl [Lauraceae] have many pharmacological activities, including antioxidant, anti-inflammatory, and immunomodulatory effects (Anderson et al., 2004). Aswar et al. showed in AR mice intranasal administration of proanthocyanidin polyphenols in AR mice reduced rubbing and sneezing, histamine, IgE, and NO levels in the serum (Aswar U. M. et al., 2015). Therefore, this phytochemical reduced the inflammatory markers in the treated mice and improved AR.

#### 3.3.3 Resveratrol

Resveratrol is a polyphenolic phytochemical found in many herbs and phytochemicals, especially in grapes, and shows many pharmacological activities, including anti-diabetes, anti-Alzheimer, anti-cancer, anti-inflammatory, anti-oxidant, and improvement of lung and liver function (Delmas et al., 2011). A study in mice showed that resveratrol significantly alleviated AR by blocking the thioredoxin-interacting protein (TXNIP) oxidative stress pathway (Zhang et al., 2020). Another study of adults with severe chronic AR found that resveratrol treatment significantly reduced nasal symptoms such as congestion, itching, sneezing, and rhinorrhea compared to a placebo-treated group, and increased quality of life in people with AR. These data suggested that resveratrol has medicinal potential (Lv et al., 2018).

#### 3.3.4 Resveratrol and β-glucan

Resveratrol is a non-flavonoid polyphenol with anti-inflammatory activity. On the other hand, β-glucan is a polysaccharide that has been noted for its anti-inflammatory and immune modulator activities (Müller et al., 1996). Del Giudice et al. demonstrated that intranasal administration of resveratrol and β-glucan to children with AR reduced allergic symptoms including itching, sneezing, rhinorrhea, and obstruction (Miraglia Del Giudice et al., 2014). This effect is probably caused by the inhibition of inflammatory pathways, including the NF-κB, IκB, and COX-2 signaling pathways (Zang et al., 2011; Mastromarino et al., 2012).

### 3.4 Terpenoids

#### 3.4.1 1,8-cineole

1,8-cineole (Eucalyptol) is a monoterpenoid that naturally occurs in volatile oils of several species such as *Peumus boldus* Molina [Monimiaceae] (Urzúa et al., 2010; Fuentes-Barros et al., 2023) 1,8-cineole inhibits the production of prostaglandins and leukotrienes, which are important causes of AR. In one study, the molecular mechanism underlying the protective effect of cineole against OVA-induced AR in mice was investigated. Compared to the untreated group, cineole significantly reduced Th2-type cytokines and OVA-specific IgE in mice with AR, improved nasal mucosal tissue damage, and relieved nasal symptoms (Liu F.-L et al., 2023).

#### 3.4.2 Glycyrrhizic acid

Glycyrrhizic acid is a triterpene glycoside found in the roots of licorice *Glycyrrhiza glabra* L. [Fabaceae]. The anti-viral, anti-oxidant, and anti-cancer activities of this triterpene glycoside have been previously confirmed (Sasaki et al., 2002; Khazraei-Moradian et al., 2017). Fouladi et al. demonstrated that the *in vitro* treatment of CD4^+^ T cells with glycyrrhizic acid reduced OX40 expression. OX40 is a receptor of the TNF family (Willoughby et al., 2017). On the contrary, glycyrrhizic acid treatment raises T-bet, and GATA3 and FOXP3 protein and gene expression as well as reduces IL-4 levels, but it has no significant effect on IFN-ϒ and IL-10 levels (Fouladi et al., 2018).

#### 3.4.3 Glycyrrhizin

Glycyrrhizin is a triterpene glycoside isolated from *G. glabra* and has pharmacological activities, including anti-oxidant, anti-inflammatory, and anti-cancer (Finney and Somers, 1958; Shibata et al., 1987). Li et al. confirmed that treatment of AR mice with glycyrrhizin reduced IgE, IL-4, IL-5, IL-6, NO, TNF-α, and NOS, and increased IgA, IgG, IL-2, and IL-12 levels in the blood as well as in nasal mucosa, reduced substance P, and raised acetylcholinesterase (AchE) activity (Li and Zhou, 2012).

#### 3.4.4 Platycodin D

Platycodin D is a triterpenoid saponin isolated from *Platycodon grandiflorum* (Jacq.) A.DC. [Campanulaceae], which exhibits many pharmacological activities, such as anti-inflammatory, anti-cancer, immune modulator, and anti-oxidant activities (Chung et al., 2008; Chun et al., 2013). Wang et al. demonstrated that treatment of human nasal epithelial cells (RPMI2650) with platycodin D reduced NF-κB, eotaxin, and GM-CSF protein levels and reduced the mRNA expression of mucin 5AC (MUC5AC), ERK, and P65 (Wang B. et al., 2016). Therefore, this phytochemical improves AR *in vitro* via inhibition of the MAPK signaling pathway and mucus production.

#### 3.4.5 Thymol

Thymol (2-isopropyl-5-methylphenol) is a phenolic monoterpene found in several plants such as oregano and thyme. Thymol exhibits multiple biological and pharmacological properties, including anti-inflammation and anti-oxidation potential, and can be utilized to treat a wide range of inflammatory conditions, including AR. Thymol was examined to see how it affects mices with OVA1-induced AR. According to the findings, thymol reduces inflammation and allergy symptoms in this AR model. Comparing the AR group with the other groups, a significant decrease in total antioxidant capacity (TAC) levels and an increase in IgE, IL-5, IL-13, and total oxidant status (TOS) levels in thymol group were observed (Kilic et al., 2019).

#### 3.4.6 Tussilagone

Tussilagone is a sesquiterpenoid isolated from Farfarae Flos, the dried flower buds of *Tussilago farfara* L. [Asteraceae], which has been used as a traditional medicine for the treatment of asthma and bronchitis because of its anti-inflammatory activity (Cheon et al., 2018). Cheon et al. demonstrated that tussilagone reduced histamine, IgE, IL-6, and TNF-α levels in AR guinea pigs *in vitro* in RBL2H3 cells, demonstrating that treatment with tussilagone reduces Lyn, Syk, NF-κB, ERK, p38, and MAPK expression (Cheon et al., 2018). Therefore, this sesquiterpenoid inhibits the NF-κB/ERK/MAPK signaling pathway, and inhibition of proinflammatory cytokine production modulates inflammation and improves AR.

#### 3.4.7 Ursolic acid

Ursolic acid (3β-hydroxy-urs-12-en-28-oic acid) is a naturally derived pentacyclic triterpenoid widely distributed in various plant foods and Chinese herbal medicines. It has been found to have numerous physiological properties, including anti-inflammatory, anticancer, bone regeneration, anti-fungal, hepatoprotective, and antioxidant effects (Seo et al., 2018). Based on these findings, Ursolic acid demonstrates potential in the management of allergic inflammation, particularly in alleviating nasal symptoms and inhibiting the expression of Th2 cytokines and eosinophilic infiltration. In a rat model of AR following exposure to particulate matter ≤2.5 μm (PM_2.5_), ursolic acid mitigates mucus secretion and tissue remodeling, while also reducing the frequency of sneezing and nasal rubbing. Furthermore, ursolic acid impedes FcεRI-mediated mast cell activation and allergic inflammation. However, the impact of ursolic acid intervention on PM_2.5_-induced AR remains unclear (Sun et al., 2021).

### 3.5 Miscellaneous compounds

#### 3.5.1 (2′*S*,7′*S*)-*O*-(2-methylbutanoyl)-columbianetin

(2′*S*,7′*S*)-*O*-(2-methylbutanoyl)-columbianetin is an active phytoalexin isolated from *Corydalis heterocarpa* var. *japonica (*Franch. and Sav.) Ohwi [Papaveraceae] extract. This plant has been used since the past as an anti-inflammatory agent for the treatment of inflammatory diseases in Korea (Kim et al., 2010). Nam et al. showed that treatment of the human leukemic cell line with (2′*S*,7′*S*)-*O*-(2-methyl butanol)-columbianetin reduced histamine and tryptase levels; reduced IL-1β, IL-6, IL-8, and TNF-α levels; inhibited the MAPK signaling pathway via inhibition of ERK, JNK, and p38 phosphorylation, and inhibited the phosphorylation of IκB-α and NF-κB. This phytochemical also reduced caspase-1 expression and activity in human leukemic cell lines *in vitro.* During the *in vivo* investigation on AR mice, it has been confirmed that treatment with (2′*S*, 7′*S*)-*O*-(2-methyl butanol)-columbianetin reduced the histamine, IgE, and IL-1β levels in serum also reduced the MIP-2 and Intercellular Adhesion Molecule 1 (ICAM-1) protein levels and eosinophils and mast cells in the nasal mucosa tissue and reduced the spleen weight and increased the IFN-ϒ levels in the spleen tissue (Nam et al., 2014). This study confirmed that (2′*S*, 7′*S*)-*O*-(2-methyl butanol)-columbianetin is a potent anti-AR compound that acts via inhibition of the MAPK/NFκB signaling pathway, inhibition of proinflammatory and Th2 cytokines, and increasing the Th1 cytokines.

#### 3.5.2 Alpha linolenic acid

α-Linolenic acid (18:3n-3) is an essential omega-3 polyunsaturated fatty acid that is found in plant seed oils and beans. Ren et al. found that α-linolenic acid reduced inflammation in mice with OVA-induced AR by regulating Th1/Th2 imbalance (Ren et al., 2022). Furthermore, Ding et al. suggested that α-linolenic acid enhances the nasal mucosal epithelial barrier function in AR by inhibiting CD4^+^ T cell differentiation via the IL-4Rα-JAK2-STAT3 pathway (Ding et al., 2023). In addition, they showed that α-linolenic acid improves nasal mucosal epithelial barrier function in AR patients.

#### 3.5.3 Alpha lipoic acid

Alpha lipoic acid (thioctic acid) is an organosulfur compound with strong antioxidant properties. It is used to regulate diabetic neuropathy and has potential applications in the treatment of other diseases associated with oxidative stress and inflammation. Nguyen et al. showed that alpha lipoic acid has a positive effect on allergic inflammation in a mouse model of AR. Alpha lipoic acid treatment improved the differentiation and function of Treg cells, which contributed to the balance of Th17/Treg expression and increased Nrf2/heme oxygenase-1 (HO-1) pathway signaling. Alpha lipoic acid administration also significantly reduces nasal symptoms, such as rubbing and sneezing, and improves the histopathology of the nose and lungs (Van Nguyen et al., 2020).

#### 3.5.4 Acetylshikonin

Acetylshikonin is a naphthoquinone isolated from purple gromwell *Aegonychon purpurocaeruleum* (L.) Holub [Boraginaceae]. The anti-inflammatory and antioxidant activities of this naphthoquinone have been confirmed previously (Singh et al., 2003; Cheng et al., 2008). Fan et al. demonstrated that in AR mice treatment with acetyl shikonin reduced the IgE and IgG1 levels but it had no effect on IgG2α levels in serum also in the nasal lavage fluid it caused a reduction in the Th2 cytokines including IL-4, IL-5, IL-13, GATA-3, and TNF-α levels also reduced the histamine levels in serum and nasal lavage fluid but it has not a significant effect on Th1 cytokines including IFN-ϒ, IL-10 and IL-12 levels (Fan X. et al., 2019). This study confirmed that acetyl shikonin exerts its anti-AR effect via inhibition of the Th2 response.

#### 3.5.5 Caffeoylxanthiazonoside

Caffeoylxanthiazonoside is an active constituent isolated from the fruit of *Xanthium strumarium* L. [Asteraceae] and studies have confirmed the oxidant anti-inflammatory and anti-asthmatic activities of caffeoylxanthiazonoside (Wu Q. et al., 2019). Peng et al. demonstrated that treatment with caffeoylxanthiazonoside reduced IgE levels in the serum, as well as nasal sneezing and scratching in mice with AR (Peng et al., 2014). In another study, it has been confirmed that treatment of AR mice with caffeoylxanthiazonoside-coated gold nanoparticles reduced the IFN-ϒ and IL-4 levels in nasal lavage fluid. Histopathological studies confirmed an improvement in AR in mice (Peng and Chen, 2019).

#### 3.5.6 D-pinitol

D-pinitol (*O*-methyl inositol) is a cyclitol found in many foods, such as soy, and legumes, and has a wide range of pharmacological activities, such as antioxidant, anti-inflammatory, anti-cancer, and anti-aging activities (Sethi et al., 2008). You et al. demonstrated that in AR mice treated with D-pinitol symptoms of improvement were seen due to Th1/Th2 response balancing such as reducing the IgE, IgG1, IL-4, IL-5, IL-13, LTC-4 and increasing the IFN-ϒ levels in nasal lavage fluid also reduce the GATA-3, STAT-6, SOCS1, TLR4, and MyD88 expression and increase the T-bet expression in the spleen tissue (You et al., 2021).

#### 3.5.7 Fucoxanthin

Fucoxanthin is a carotenoid found in brown seaweed that has anti-inflammatory and antioxidant effects. A study in male BALB/c mice showed that fucoxanthin could successfully prevent the development of OVA-induced AR. This study investigated the effect of fucoxanthin on allergic mice and discovered that allergic reactions, such as rubbing and sneezing, reflected the effect of fucoxanthin in both the stimulated and treated groups. The mean histological scores were different between the OVA-treated and fucoxanthin-treated groups in terms of ciliary loss, eosinophil infiltration, and other factors. The modulatory effect of fucoxanthin on AR was confirmed by the lipid profile (malondialdehyde). In addition, reduced IgE and histamine levels were observed in the fucoxanthin-treated groups (Li et al., 2019).

#### 3.5.8 Geniposide

Geniposide is the main iridoid glucoside of *Gardenia jasminoides* J.Ellis [Rubiaceae] that is used for the treatment of inflammatory diseases such as colitis and rheumatoid arthritis (Shin et al., 2018). Zhang et al. demonstrated that treatment of AR mice with geniposide reduced IgE, IL-4, IL-5, and IL-17 levels and increased the IL_2 and IFN-ϒ levels in serum, and reduced CD4^+^ T cells and Foxp3 cell population in the spleen tissue (Zhang et al., 2019).

#### 3.5.9 Magnolol

Magnolol is a lignan derived from *Magnolia* spp. [Magnoliaceae]. It has pharmacological activities such as anti-inflammatory, anti-allergic, anti-asthmatic, and immunomodulatory activities (Huang et al., 2019). Phan et al. demonstrated that *in vitro* treatment of HEK293T cells and Calu-3 cells with magnolol inhibits ORAI1 (which causes T-lymphocyte and mast cell activation and initiates allergy) and anoctamin 1 (ANO1) (which causes mucin secretion and allergic inflammation) in HEK293T cells and chloride secretion in the Calu-3 cells also reduced IL-2 levels in T-lymphocytes. In an *in vivo* study on AR mice, intranasal administration of magnolol reduced IL-13 levels in the nasal mucosa and eosinophil infiltration in the nasal tissue, and also reduced sneezing and rubbing in AR mice (Phan et al., 2022). Therefore, reducing the levels of inflammatory markers in this phytochemical can improve AR.

#### 3.5.10 Mangiferin

Mangiferin (1,3,6,7-tetrahydroxyxanthone-C2-β-D-glucoside) is a C-glucosyl xanthone predominantly isolated from the mango tree. Mangiferin has anti-allergic, anti-inflammatory, and antioxidant properties related to AR. Mangiferin effectively inhibited OVA-induced nasal allergic symptoms, as evidenced by the reduction in rubbing and sneezing scores. In an AR model, mangiferin reduced ciliary loss, vascular congestion in the lamina, goblet cell elevation, and eosinophil infiltration, indicating its anti-inflammatory properties (Piao et al., 2020). Mangiferin exerts antioxidant effects by modulating the Th1/Th2/Th17 pathway. Consequently, Mangiferin has been shown to reduce AR by activating the Nrf2/HO-1/NF-κB signaling pathways (Piao et al., 2020).

#### 3.5.11 Total glucosides of paeony

The total glucosides of paeony derived from the roots of *Paeonia lactiflora* Pall. [Paeoniaceae] show pharmacological activities, such as anti-oxidant, anti-inflammatory, anti-pain, and immune modulators (Jiang et al., 2020). Jin et al. demonstrated that treatment of AR mice with total glycosides of paeony reduced TGF-β increased Smad7 expression in the nasal tissue and reduced IgE levels in serum, sneezing, and rubbing. In the histopathological evidence of nasal tissue, this phytochemical treatment reduced the number of eosinophils, gablet cells, and collagen fibers. It also reduced malondialdehyde (MDA) and increased glutathione (GSH), catalase (CAT), and superoxide dismutase (SOD) levels in the serum. Reduced apoptosis by reducing Apoptosis regulator bcl-2-like protein 4 (BAX) and Caspase-3 levels and increasing B-cell lymphoma 2 (Bcl2) expression in the serum and nasal tissues. also reduced IL-4, IL-5, IL-17, and IFN-ϒ levels in serum (Jin and Zhang, 2022). This study confirmed that total glycosides of paeony improve AR by modulating inflammation, apoptosis, and the SMAD/TGF-β signaling pathway.

Supplementary Table 1 provides comprehensive details of the impact of phytochemicals on cellular, molecular, and external markers that are effective in improving AR. The ↑ and ↓ symbols show the effect of phytochemicals on the increase and decrease of the effective factors in AR, respectively.

## 4 Discussion

The findings of this study indicate that various phytochemicals, including flavonoids (such as apigenin, baicalin, cirsilineol, diosmetin, hesperidin, kaempferol, luteolin, morin, myricetin, naringenin, quercetin, skullcapflavone II, and tangeretin), alkaloids (including berberine, dictamenine, ellipticine, higenamine, *N,N*-dicoumaroylspermidine, piperine, sinomenine, and warifteine), terpenoids (particularly 1,8-cineole, glycyrrhizic acid, menthol, glycyrrhizin, platycodin D, thymol tussilagone, and ursolic acid), as well as various miscellaneous compounds (such as (2′S,7′*S*)-*O*-(2-methylbutanoyl)-columbianetin, alpha-linolenic acid, alpha-lipoic acid, acetylshikonin, caffeoylxanthiazonoside, D-pinitol, fucoxanthin, magnolol, and mangiferin), exhibit significant anti-inflammatory and antihistaminic properties. These compounds mitigate tissue damage and alleviate symptoms associated with AR by inhibiting inflammatory mediators, oxidative stress, and apoptosis. The mechanisms through which these phytochemicals exert their effects include the inhibition of the MAPK/NF-κB signaling pathway (notably by (2′*S*,7′S)-*O*-(2-methylbutanoyl)-columbianetin, tussilagone, platycodin D, skullcapflavone II, and diosmetin), the inhibition of IκB and COX-2 signaling pathways (as seen with resveratrol and β-glucan), the suppression of the STAT6/SOCS1 and GATA3/T-bet signaling pathways (mediated by morin), and the inhibition of the TLR4/MyD88/NF-κB signaling pathway (as demonstrated by apigenin). These pathways are integral to the therapeutic potential of these phytochemicals in the management of AR.

Allergic rhinitis results from an imbalance in the functioning of the immune system, which leads to an imbalance in the Th1/Th2 response, resulting in an increase in Th2 cytokines and a decrease in Th1 cytokines. This systematic study showed that phytochemicals inhibit the release or activity of mast cells, such as histamine, inhibit the secretion of Th2 cytokines, such as IL-4, IL-5, IL-13, and GATA-3, and reduce IgE levels as well as macrophages and lymphocytes. can reduce inflammation and thus reduce the symptoms of AR (Guo and Liu, 2013). One of the mechanisms stated in the present study that can improve AR is through the antioxidant system. It has been reported that ROS increases inflammation by activating the ERK and NF-κB pathways. Phytochemicals reduce tissue damage and inflammation with their antioxidant activity and modulate apoptosis via modulating the activities of Bax, Bcl2, and caspase (Jin and Zhang, 2022).

**FIGURE 2:**
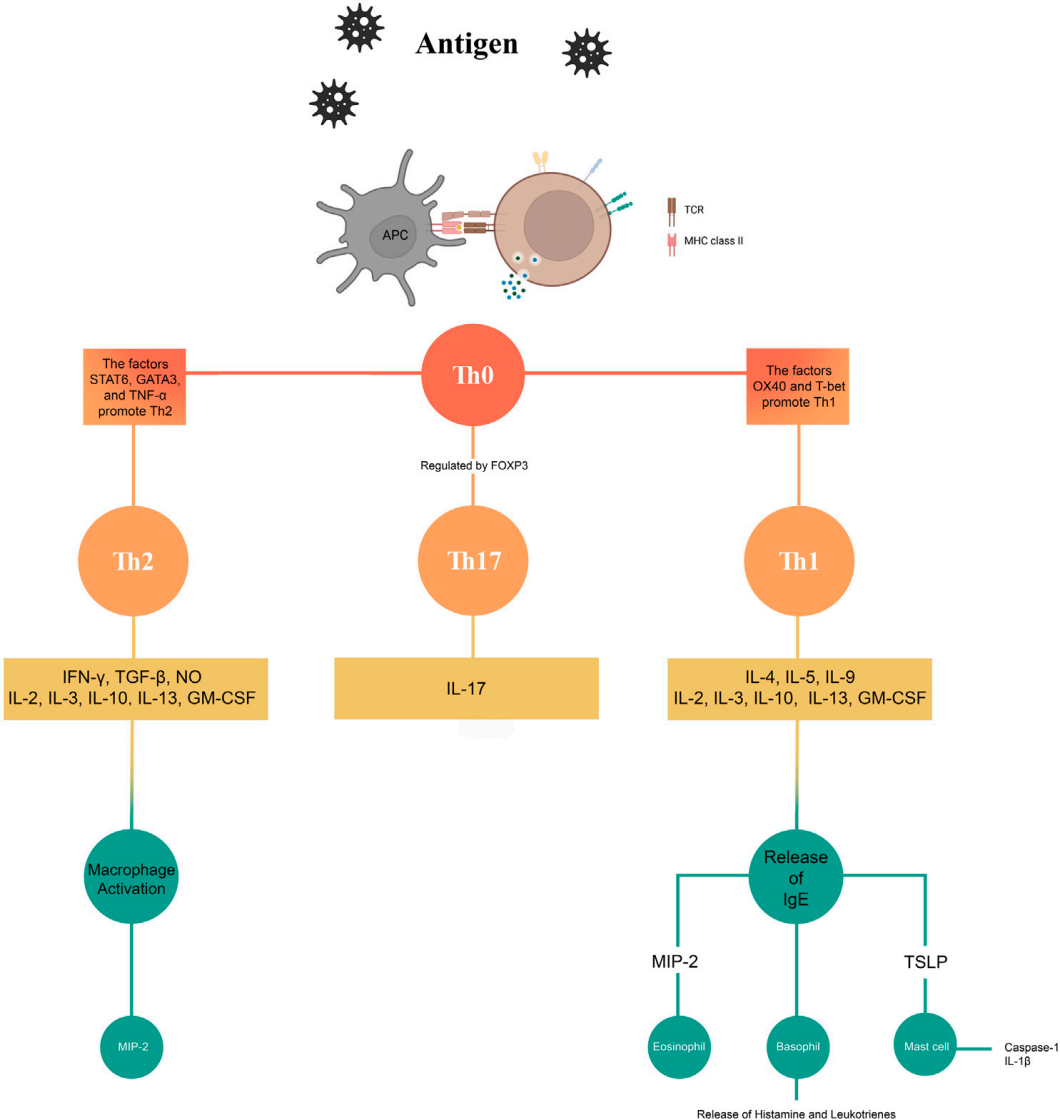
Effects of phytochemicals on the mechanisms of allergic rhinitis.

Figure 2 shows a summary of the effects of the phytochemicals investigated in this study on the mechanisms and pathways involved in AR.

Flavonoids, polyphenols, and terpenoids, which are phytochemicals combine with lymphocytes in order to regulate their function as a way of promoting the equilibrium state of immune system. Flavonoids like quercetin have been found to enhance natural killer cells’ activity and stimulate macrophage phagocytosis hence strengthening innate immunity (Hoskin and Coombs, 2022; Maheshwari et al., 2022).

Additionally, such substances can affect inflammatory pathways by changing signaling cascades, for example, JAK/STAT pathway that is important for cell–cell communication during immune response. These compounds act like adaptogens where they either enhance or suppress immune capacity depending on the situation thereby maintaining homeostasis (Bland, 2021).

Also, phytochemicals have great anti-oxidant properties since they scavenge ROS thus reducing oxidative stress in cells which is crucial for cell integrity and functioning (Hoskin and Coombs, 2022). Their ability to change gene expression through epigenetic modifications underscores their potential use in therapy particularly for treating diseases connected with immune dysfunction and improving body health in general (Behl et al., 2021). In addition, incorporating phytochemicals into dietary practices shows promise as an avenue to support immunological resilience while addressing oxidative stress-related disorders (Maheshwari et al., 2022).

Phytochemicals from medicinal plants show promise as alternative treatments for AR, but more research is needed to establish their efficacy compared to conventional therapies. While some studies suggest phytochemicals may improve symptoms like rhinorrhea and nasal itchiness, there is no clear evidence they outperform placebo or antihistamines in overall symptom relief (Lim et al., 2024). Phytochemicals appear to work by suppressing IgE, cytokines, histamine, and eosinophils (Rahim et al., 2021). However, the safety and side effect profile of most phytochemicals for AR is still unclear. More high-quality clinical trials are necessary to determine if phytochemicals can be recommended as first-line or adjunct treatments to intranasal corticosteroids, antihistamines, and other standard therapies. Patients’ preferences and quality of life should be considered when choosing between conventional and herbal treatments (Lim et al., 2024).

The present treatments, including antihistamines and corticosteroids, often have negative side effects thus suggesting the need for safer options. Scientific studies affirm that natural substances can relieve AR symptoms through the modulation of inflammatory mediators such as interleukins and TNFα, besides governing oxidative stress pathway as well. This discovery indicates that clinical practice could be improved by integrating natural products into treatment plans thus leading towards new physiological approaches in AR control (Lim et al., 2024; Park et al., 2024). More trials must be conducted on larger populations so as to confirm their safety and efficacy in this regard (Lim et al., 2024).

Some limitations have been addressed with suggestions for future studies in this article. Firstly, most of the studies inspected in this review tended to have small sample sizes, which limits the applicability of the results. Moreover, this difference of the types of plants made it indecisive about their effectiveness and innocuousness among different communities. Moreover, these products were observed to lack mechanisms that could explain their activities; hence there is a need for more detailed research on its cellular signaling pathways as well as its pharmacological targets. Furthermore, there are no long-term investigations showing how chronic users should benefit from such natural remedies considering the possible harm they may cause when combined with other types of medication. There is also another aspect regarding standardization of natural product research methodologies so that reliability and consistency can be achieved. Future clinical trials should target large multicentre settings and determine whether there is an additive effect between natural products and existing treatments against AR. This would offer a holistic approach towards AR management by improving therapeutic strategies leading to enhanced patient outcomes.

With the potential to establish lasting tolerance and avoid diseases progression, allergen immunotherapy is becoming an interesting alternative to symptomatic drugs (Gomes, 2014; Reinwald et al., 2022).

Conversely, conventional immunotherapy regimens may be time consuming, costly and carry the danger of increased reactogenicity This has led scientists to investigate new protocols such as the use of natural compounds in the treatment of allergic reactions like alkaloids and flavonoids (Sakshi et al., 2023). Such substances have shown anti-inflammatory, antihistaminic and immunomodulating activities during preclinical studies (Sakshi et al., 2023).

Our findings are part of a growing body of evidence suggesting that natural products may represent a reasonable option for allergy immunotherapy. The improvements seen in symptom scores with concomitant reductions in medication use are consistent with sublingual allergen immunotherapy (SLIT)/oral immunotherapy (OIT) studies using standardized allergen extracts (Sakshi et al., 2023).

However, it is necessary to undertake larger placebo-controlled trials for further assessment of efficacy and safety profile of these natural product-based immunotherapies. Combining natural compounds with adjuvants or using them as an adjunct to conventional treatment are promising strategies to optimize outcomes (Sakshi et al., 2023). Therefore, there is need for further research into this area so as to provide more affordable and acceptable interventions for AR as well as other atopic disorders.

## 5 Conclusion

We conclude from this systematic review that phytochemicals improve the symptoms of AR by affecting antioxidant, anti-inflammatory, and anti-apoptotic factors and signaling pathways. Therefore, we hope that these phytochemicals can be used as auxiliary treatments to improve allergic diseases, especially AR.

## Figures and Tables

**Table:** 

**AchE**	acetylcholinesterase
**AKT1**	serine/threonine kinase 1
**AR**	Allergic rhinitis
**ANO1**	anoctamin 1
**APCs**	antigen-presenting cells
**AR**	allergic rhinitis
**BAX**	bcl-2-like protein 4
**Bcl2**	B-cell lymphoma 2
**Calu-3**	cultured human airway epithelial cells
**CAT**	catalase
**CD4+T**	CD4 T lymphocytes cells
**c-JUN**	transcription factor Jun
**COX-2**	cyclooxygenase 2
**EoL-1**	Eosinophilic leukemia cell line-1
**ERK**	extracellular signal-regulated protein kinase
**FcεRI**	high-affinity IgE receptor
**Foxp3**	forkhead box protein P3
**GATA3**	GATA binding protein 3
**GM-CSF**	granulocyte-macrophage colony-stimulating factor
**GSH**	glutathione
**HEK293T cells**	human embryonic kidney 293 cells
**HO-1**	heme oxygenase-1
**ICAM-1t**	intercellular adhesion molecule 1
**IDST**	intradermal (intracutaneous) skin tests
**IFNγ**	interferon-γ
**IgE**	immunoglobulin E
**IκB**	inhibitor of NFκB
**IL-1β**	interleukin-1β
**IL-4**	interleukin 4
**IL-5**	interleukin 5
**IL-6**	interleukin 6
**IL-13**	interleukin 13
**JAK2**	janus kinase 2
**LTC4**	Leukotriene C4
**Lyn**	Tyrosine-protein kinase Lyn
**mRNA**	messenger RNA
**MAPKs**	mitogen-activated protein kinases
**MDA**	malondialdehyde
**MIP-2**	Macrophage inflammatory protein-2
**MUC5AC**	mucin 5AC
**NF-κB**	Nuclear factor-kappa B
**NO**	Nitric oxide
**NOS**	nitric oxide species
**OIT**	oral immunotherapy
**OVA**	ovalbumin
**OX40**	antigen CD134
**p-ERK1/2**	phospho-ERK1/2
**PGD2**	prostaglandin D2
**PM2.5**	particulate matter 2.5 μm
**ROS**	reactive oxygen species
**RORγt**	thymus or t isoform of the retinoic acid-related orphan receptor gamma
**RPMI2650**	human nasal epithelial cells
**SLIT**	sublingual allergen immunotherapy
**SMAD**	suppressor of mothers against decapentaplegic
**SIRT1**	NAD-dependent deacetylase sirtuin-1
**SOCS**	suppressor of cytokine signaling
**SOCS1**	suppressor of cytokine signaling 1
**SOD**	Superoxide dismutase
**SPT**	skin prick test
**STAT6**	signal transducer and activator of transcription 6
**SYK**	spleen tyrosine kinase
**TAC**	total antioxidant capacity
**T-bet**	T box expressed in T cells
**TGF-β**	tumor growth factor β
**Th1**	Type 1 helper cells
**Th2**	Type 2 helper cells
**Th17**	Type 17 helper cells
**TLRs**	Toll-like receptors
**TLSP**	thymic stromal lymphopoietin
**TNFα**	tumor necrosis factor α
**TOS**	total oxidant status
**Treg**	regulatory T cells
**TXNIP**	thioredoxin interacting protein
**VIP**	vasoactive intestinal peptide
