# Supplementary material for: Natural products for the treatment of allergic rhinitis: focus on cellular signaling pathways and pharmacological targets
Source: Front Pharmacol. 2024 Sep 30;15:1447097. doi: 10.3389/fphar.2024.1447097 (PMC11472003; doi:10.3389/fphar.2024.1447097)
Supplement: Supplementary file 1 [file Table1.pdf]

Supplementary Table 1. Effective phytochemicals in the treatment of allergic rhinitis

Phytochemical/extract	Model	Animal/Cell	Optimal dose	Outcomes	References
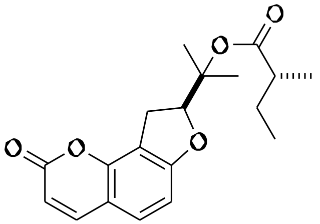 (2′S, 7′S)-*O*-(2-methylbutanoyl)-columbianetin	OVA	*In vivo*: Mouse<br><br>*In vitro*: human leukemic cell line	*In vivo*: 10 mg/kg<br><br>*In vitro*: 10 μg/mL	↓Histamine, ↓Tryptase, ↓IL-1β, ↓IL-6, ↓IL-8, ↑IFN-ϒ, ↓TNF-α, ↓ERK, ↓JNK, ↓p38, ↓IκBα, ↓NF-κB, ↓IgE, ↓MIP-2, ↓ICAM-1, ↓Spleen weight	(Nam et al., 2014)
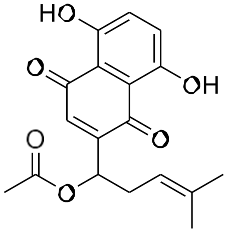 Acetylshikonin	OVA	*In vivo*: Mouse	80 mg/kg	↓IgE, ↓IgG1, ↓IL-4, ↓IL-5, ↓IL-13, ↓GATA-3, ↓TNF-α, ↓Histamine	(Fan et al., 2019a)
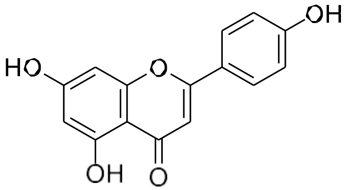 Apigenin	OVA	*In vivo*: Mouse<br><br>*In vitro*: HMC-1 cells	100-200 mg/kg	↓TLR4/MyD88/NF-κB pathway, ↓IgE, ↓IgG1 and ↓IgG2a, ↓β-hexosaminidase, ↓histamine .↓ECP ↓Th2, ↓Th1, ↓Th1/Th2 cells	(Chen et al., 2020)

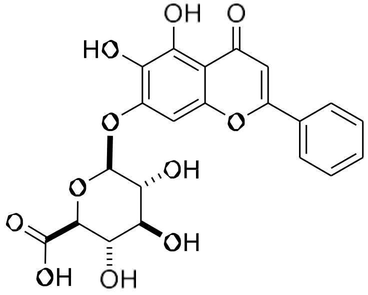<br>Baicalin	OVA	*In vivo:* Rat	200 mg/kg	↓IgE, ↓Histamine, ↓IL-4, ↓IL-1β, ↓IL-6, ↓TNF-α, ↓Nasal sneezes	(Chen et al., 2019)
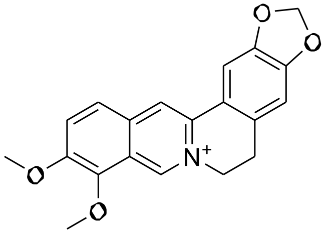<br>Berberine	House dust mite	*In vivo:* Mouse	10 μg/mL	↓nasal rubbing, ↓sneeze ↓IgE, ↓GATA-3,	(Kim et al., 2015)
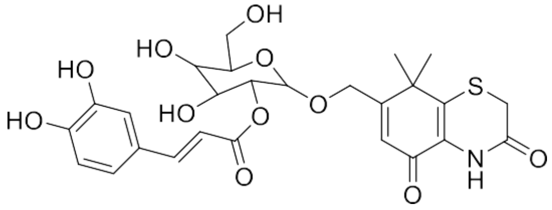<br>Caffeoylanthiazonoside	Acetic acid	*In vivo:* Mouse	20 mg/kg	↓IgE, ↓Nasal ↓sneezes, ↓Nasal ↓scratching	(Peng et al., 2014)
	OVA	*In vivo:* Mouse	10 ppm	↓IFN-ϒ, ↓IL-4	(Peng et al., 2014)
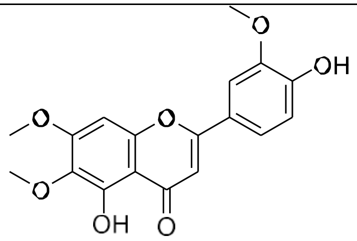<br>Cirsilineol	OVA	*In vivo:* Mouse	10 and 20 mg/ kg	↓sneezing, ↓nasal rubbings, ↓IgE, ↓PGD2, ↓LTC4 status, ↓IL-4, ↓IL-5, ↓IL-6, ↓IL-33 , ↓TNF-α<br>↓ROS, ↓MDA, ↑SOD	(Li et al., 2021)

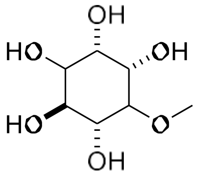<br>D-pinitol	OVA	*In vivo*: Mouse	20 mg/kg	↓IgE, ↓IgG1, ↓IL-4, ↓IL-5, ↓IL-13, ↓LTC-4, ↓GATA-3, ↓STAT-6, ↓SOCS1, ↓TLR4, ↓MyD88, ↑T-bet, ↑IFN-ϒ	(You et al., 2021)
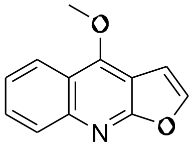<br>Dictamnine	OVA	*In vivo:* Mouse<br><br>*In vitro:* LAD2 human MCs	*In vivo:* 200 μg/mouse<br><br>*In vitro:* 1-200 μM	↓FcεRI<br>↓tyrosine kinase LYN<br>↓phosphorylation of PLCγ1, IP3R, PKC, Erk1/2, and Akt	(Liu et al., 2023b)
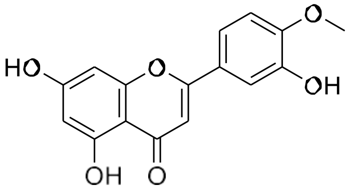<br>Diosmetin	OVA	*In vivo:* Mouse	200 μL (0.5 mg/kg)	↓rubbing, ↓sneezing, ↓discharge<br>↓eosinophil and mast cell infiltration in nasal mucosa<br>↓Th1/Th2 imbalance<br>↑SIRT1<br>↓ NF-κB pathway	(Hu and Peng, 2023)
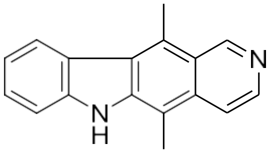<br>Ellipticine	OVA	*In vivo:* Mouse	5-15 mg/kg	↓IgG1, ↓TNF-α , ↓ IL-1β ↓MIP-2, ↓COX-2, ↓NF-κB	(Wang et al., 2021)
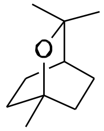	OVA	*In vivo:* Mouse	30 mg/kg	↓rubbing, ↓sneezing, ↓discharge, ↓Th2-type, ↓VA-specific IgE (OVA-	(Liu et al., 2023a)

Eucalyptol (1,8-cineole)				sIgE), ↓leukotrienes (LTs) prostaglandin	
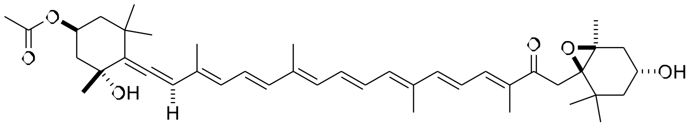<br>Fucoxanthin	OVA	*In vivo:* Mouse		↓Rubbing<br>↓sneezing<br>↓eosinophil ↓Ig E<br>↓histamine<br>↓MDA<br>↓NF-κB p65<br>↓STAT 3	(Li et al., 2019)
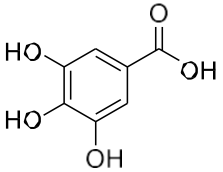<br>Gallic acid	OVA	*In vivo:* Mouse	80 mg/kg	↓Rubbing, ↓Sneezing, ↓Neutrophils, ↓Lymphocytes, ↓Macrophages, ↓IgE, ↓IgG1, ↓IL-4, IL-5, ↓IL-13, ↓IL-17, ↓RORϒt, ↑IgG2α, ↑IL-12, ↑IFN-ϒ	(Fan et al., 2019b)
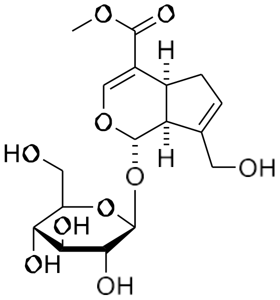<br>Geniposide	OVA	*In vivo:* Mouse	7 mg/kg	↓Scratching, ↓Sneezing, ↓IgE, ↓IL-4, ↓IL-5, ↓CD4+T cells, ↓IL-17, ↓Foxp3, ↑IL-2, ↑IFN-ϒ	(Zhang et al., 2019)

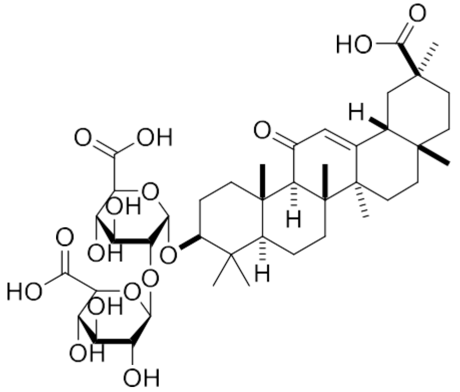<br>Glycyrrhizic acid	-	*In vitro:* CD4+ T cells	200 µM	↓OX40, ↓IL-4, ↑T-bet, ↑GATA-3, ↑Foxp3	(Fouladi et al., 2018)
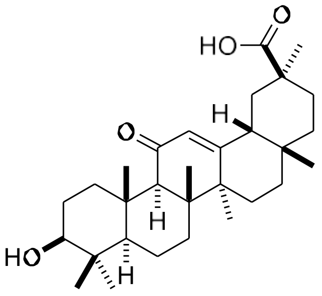<br>Glycyrrhizin	OVA	*In vivo*: Mouse	30 mg/kg	↓IgE, ↓IL-4, ↓IL-5, ↓IL-6, ↓NO, ↓TNF-α, ↓NOS, ↓Substance p, ↑IgA, ↑IgG, ↑IL-2, ↑IL-12, ↑Ach E activity	(Li and Zhou, 2012)
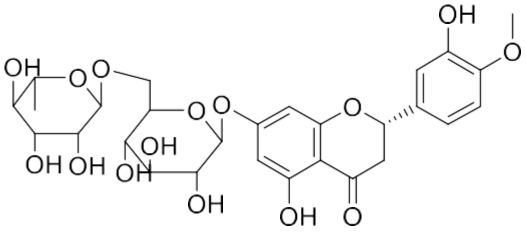<br>Hesperidin	OVA	*In vivo*: Mouse	100 mg/kg	↓Nasal symptom, ↓Ig-E, ↓ IL-5, ↓IL-13, ↓TOS	(Kilic et al., 2019)

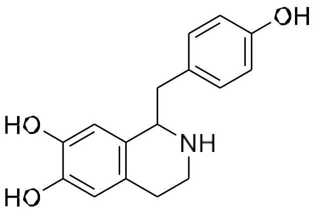 Higenamine	OVA	*In vivo:* Mouse	30, 60, and 120 mg/kg	↓rubbing, ↓sneezing, ↓IgE, ↓histamine, ↓IL-4, ↓Th1/Th2 imbalance ↓mRNA expression (IL)-6, and IL-8, ↓MUC5AC, ↓phosphorylation of NF-κB	(Wei et al., 2021)
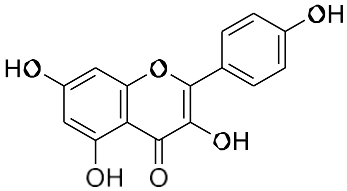 Kaempferol	*In vivo:* OVA<br>*In vitro:* GM-CSF	*In vivo:* mouse<br>*In vitro:* Eol-1 cells	*In vivo:* 20 mg/kg<br>*In vitro:* 20 μg/mL	↓IL-8, ↓Caspase-1, ↓Spleen weight, ↓IgE, ↓IL-4, ↓Histamine, ↓IL-32, ↓TLSP, ↓MIP-2, ↓ICAM-1,↓Cox-2,↑IFN-ϒ	(Oh et al., 2013)
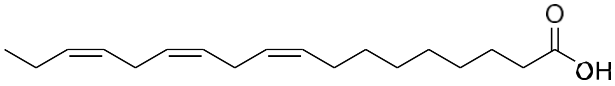 α-Linolenic acid	OVA	*In vivo:* Mouse	2000 mg/kg	↓rubbing, ↓sneezing, ↓discharge ↓IgE, ↓IL-4 ↓mRNA expression levels of IL-6 and IL-1β ↓Th1/Th2 imbalance ↑mRNA expression levels of T-bet and STAT1 ↓GATA3, ↓STAT6	(Ren et al., 2022)
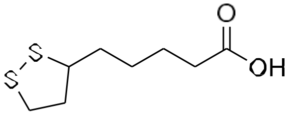	OVA	*In vivo:* Mouse	2, 10, and 50 mg/kg	↓Rubbing, ↓sneezing, ↓IgE ↓IgG1 , ↑IL-10 , ↑ Foxp3, ↓ Th17, ↓IL-17, ↓STAT3, ↓RORγ,	(Van Nguyen et al., 2020)

α-lipoic acid					
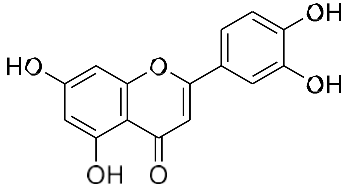<br>Luteolin	House dust mite	*In vivo:* Mouse<br><br>*In vivo:* Mouse<br><br>*In vitro:* Peripheral blood mononuclear cells	10 mg/kg or 30 mg/kg<br><br>*In vivo:* 30 mg/kg<br><br>*In vitro:* 10 μg/mL	↓nasal scratching, ↓sneezing, ↓IgE, ↓infiltration of eosinophils, ↓mucus secretion of nasal epithelium<br><br>↓IgE, ↓IgG, ↓IL-4, ↓IL-10, ↓STAT-6, ↓GATA-3, ↑IFN-ϒ, ↑IL-17, ↑IgG2α	(Liang et al., 2020)<br><br>(Dong et al., 2021)
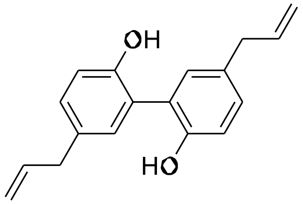<br>Magnolol	OVA	*In vivo:* Mouse<br><br>*In vitro:* HEK293T cells and Calu-3 cells	*In vivo:* 300 μM<br><br>*In vitro:* 30 μM	↓ORAI1,↓ANO1, ↓Chloride secretion, ↓IL-2, ↓IL-13, ↓Sneezing, ↓Rubbing, ↓Eosinophils	(Phan et al., 2022)

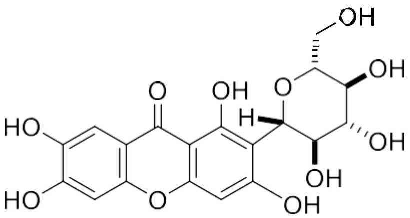<br>Mangiferin	OVA	*In vivo:* Mouse<br><br>*In vivo:* Mouse	25 mg/kg<br><br>20 mg/kg	↓sneezing,↓ rubbing, ↓ MDA, ↓STAT3, ↓NF-κBp65<br><br>↓nasal symptoms, ↓nasal mucosa inflammation ↓Th2/Th17 cytokines, ↓HO-1/Nrf2 pathways, ↓NF-κB signaling pathways	(Piao et al., 2020; Wang et al., 2020)
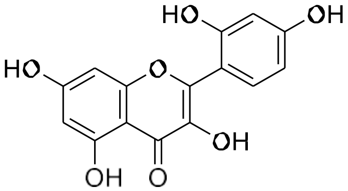<br>Morin	OVA	*In vivo:* Mouse	0, 30 and 100 mg/kg	↓rubbing, ↓sneezing, ↓discharge, ↓histamine, ↓IgE, ↓IgG1, ↓β-hexosaminidase, ↓nasal lavage fluid Th2 cytokines (IL-4, IL-5, IL-13, and IL-17),<br>↓nasal lavage fluid Th1 cytokines (IFN-γ), ↓GATA3, ↓p-STAT6, ↓SOCS1	(Liang et al., 2019)
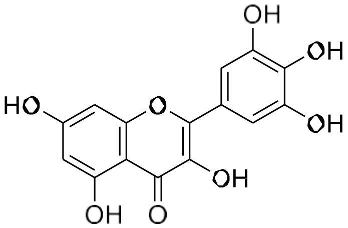<br>Myricetin	OVA	*In vivo:* Mouse	100 and 200 mg/kg	↓rubbing, ↓sneezing, ↓discharge<br>↓Histamine, ↓IgE, ↓ IgG1, ↓β-hexosaminidase,<br>↓Th1/Th2 imbalance<br>↓leukotriene C4<br>↑T-box<br>↓GATA 3, ↓NF-κB,	(Shi et al., 2023)

				↓1κB-α	
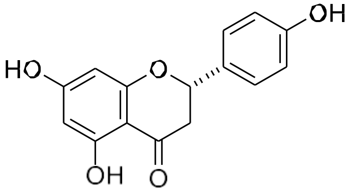<br>Naringenin	OVA	*In vivo:* Rat	100 mg/kg	↓sneezing, ↓nasal itching, ↓IgE, ↓IL4 , ↓IL5	(Şahin et al., 2021)
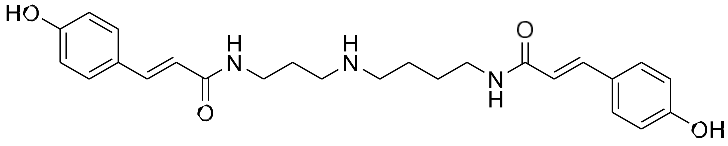<br>*N*,*N*-dicoumaroylspermidine	OVA	*In vivo:* Mouse	50 or 100 µg	↓β-hexosaminidase, ↓IL-3, ↓IL-4, ↓IL-13,	(Le et al., 2022)
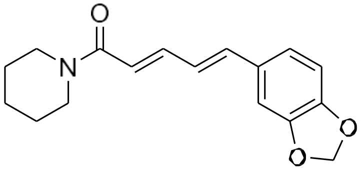<br>Piperine	OVA	*In vivo:* Mouse	40 mg/kg	↓Spleen weight, ↓NO, ↓Histamine, ↓IL-1β, ↓IgE, ↓IL-6	(Aswar et al., 2015b)
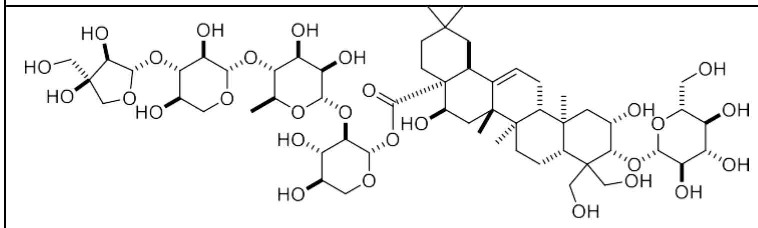<br>Platycodin D	IL-13	*In vitro:* Human nasal epithelial cell line (RPMI2650)	50 µM	↓Eotaxin, ↓GM-CSF, ↓Muc5ac,↓P65	(Wang et al., 2016a)

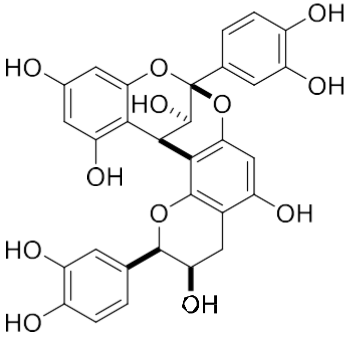 A-type-procyanidin	OVA	*In vivo:* Mouse	30 µg/kg	↓Rubbing, ↓Sneezing, ↓Histamine, ↓IgE, ↓NO	(Aswar et al., 2015a)
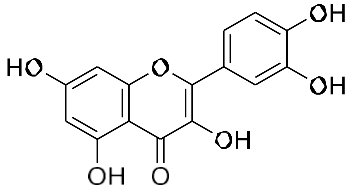 Quercetin	OVA	*In vivo:* Rat	80 mg/kg	↓Cox-2, ↓VIP	(Sagit et al., 2017)
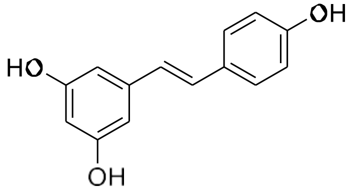 Resveratrol	OVA	*In vivo:* Mouse<br><br>Clinical trial	200 mg or 400 mg<br><br>100 µL/spray	↓sneezing, ↓nasal rubbing ↓IgE, ↓PGD2, ↓LTC4, ↓ECP, ↓IL-4, ↓IL-5, ↓IL-6, ↓IL33, ↓TNF-a, ↓eosinophil, ↓TXNIP-oxidative stress pathway<br>↓nasal symptoms, ↓IgE, ↓IL-4, ↓TNF-α,	(Lv et al., 2018; Zhang et al., 2020)
Resveratrol and β-glucan	-	Clinical trial	100 µL/spray	↓Itching, ↓Sneezing, ↓Rhinorrhea, ↓Obstruction	(Miraglia Del Giudice et al., 2014)

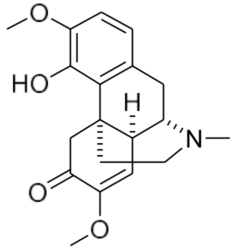 Sinomenine	OVA	*In vivo:* Mouse	100 mg/kg	↓Rubbing, ↓Sneezing, ↓Eosinophils,↓IgE, ↓IL-4,↓IFN-ϒ, ↑TGF-β	(Chen et al., 2017)
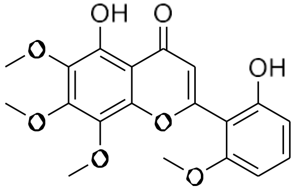 Skullcapflavone Ⅱ	OVA	*In vivo:* Mouse	10 mg/kg	↓Eosinophils, ↓PAS+ cells, ↓Mast cells, ↓IgE, ↓IgG1, ↓TNF-α, ↓IL-4, ↓IL-13, ↓GATA-3, ↓Histamin, ↓NF-κB, ↑IκB, ↑IL-12	(Bui et al., 2017)
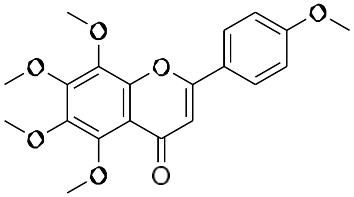 Tangeretin	OVA	*In vivo:* Mouse	50 mg/kg	↓inflammation, ↓IgE, ↑CD4 + CD25 + FOXP3 + Treg cell	(Xu et al., 2019)
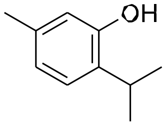 Thymol	OVA	*In vivo:* Mouse	100 mg/kg	↓Nasal symptom, ↓Ig-E, ↓ IL-5, ↓IL-13, ↓TOS	(Kilic et al., 2019)
Total glycosides of paeony	OVA	*In vivo:* Mouse	120 mg/kg	↓TGF-β, ↓IgE, ↓Sneezing, ↓Rubbing, ↓Eosinophils, ↓Goblet cells, ↓Collagen fibers,	(Jin and Zhang, 2022)

				↓MDA, ↓Bax, ↓Caspase-3, ↓IL-4, ↓IL-5, ↓IL-17, ↓IFN-ϒ, ↑Smad-7, ↑GSH, ↑CAT, ↑SOD	
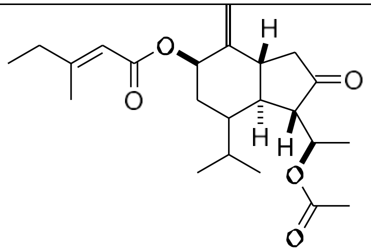<br>Tussilagone	*In vivo:* OVA<br><br>*In vitro:* IgE	*In vivo:* Guinea pig<br><br>*In vitro:* RBL2H3 cells	*In vivo:* 50 mg/kg<br><br>*In vitro:* 500 µM	↓Histamine, ↓IgE, ↓IL-6, ↓TNF-α, ↓Lyn, ↓Syk, ↓NF-κB, ↓ERK, ↓p38, ↓MAPK	(Cheon et al., 2018)
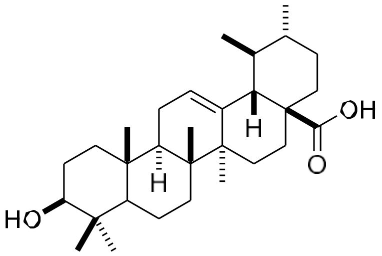<br>Ursolic Acid	$PM_{2.5}$	*In vivo:* Rat	20 mg/kg	↓mucus secretion, ↓sneezes, ↓nasal rubs, ↓FcεRI-mediated mast cell activation	(Sun et al., 2021)
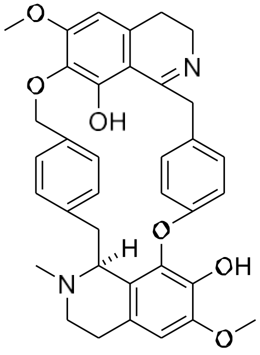<br>Warifteine	OVA	*In vivo:* Mouse	2 mg/kg	↓IgE, ↓Mucus, ↓Mast cells	(Vieira et al., 2018)
